# Beyond BMI: Roles of SPISE, Central Adiposity and Body Composition in Pediatric Brain Tumor Survivors

**DOI:** 10.3390/cancers18213494

**Published:** 2026-10-29

**Authors:** Amanda Casirati, Giacomo Tantari, Giuseppe D’Annunzio, Alessandro Suppa, Marco Del Monte, Margherita Di Jorgi, Valentina Petrone, Marta Panciroli, Gianluca Piccolo, Marta Molteni, Antonia Ramaglia, Anna Elsa Maria Allegri, Alessia Angelelli, Daniela Fava, Alessandro Naim, Flavia Napoli, Caterina Tedesco, Mohamad Maghnie, Natascia Di Iorgi

**Affiliations:** 1 Pediatric Clinic and Endocrinology Unit, IRCCS Istituto Giannina Gaslini, 16147 Genoa, Italy; amandacasirati@gaslini.org (A.C.); giacomotantari@gaslini.org (G.T.); giuseppedannunzio@gaslini.org (G.D.); martapanciroli@gaslini.org (M.P.); annaallegri@gaslini.org (A.E.M.A.); alessiaangelelli@gaslini.org (A.A.); danielafava@gaslini.org (D.F.); alessandronaim@gaslini.org (A.N.); flavianapoli@gaslini.org (F.N.); caterinatedesco@gaslini.org (C.T.); mohamadmaghnie@gaslini.org (M.M.); 2 Department of Neuroscience, Rehabilitation, Ophthalmology, Genetics, Maternal and Child Health, University of Genoa, 16132 Genoa, Italy; alessandro.suppa93@gmail.com (A.S.); marco.delmonte.md@gmail.com (M.D.M.); margheritadijorgi@gmail.com (M.D.J.); valentina.97.vp@gmail.com (V.P.); 3 Neurooncology Unit, IRCCS Istituto Giannina Gaslini, 16147 Genoa, Italy; gianlucapiccolo@gaslini.org (G.P.); martamolteni@gaslini.org (M.M.); 4 Neuroradiology Unit, IRCCS Istituto Giannina Gaslini, 16147 Genoa, Italy; antoniaramaglia@gaslini.org

**Keywords:** SPISE, central adiposity, body composition, childhood cancer survivors, brain tumor, hypothalamic–pituitary dysfunction, insulin sensitivity

## Abstract

Background/Objectives. Pediatric brain tumor survivors (PBTS) are at increased risk of cardiometabolic complications due to tumor- and treatment-related hypothalamic–pituitary (HP) damage and adverse body composition. We evaluated associations of the Single-Point Insulin Sensitivity Estimator (SPISE) with central adiposity, DXA-derived body composition, metabolic abnormalities, and HP involvement. Methods. In this retrospective study, 119 PBTSs (median age, 12.7 years; median time since therapy, 2.4 years) were evaluated. SPISE, BMI, waist-to-height ratio (WHtR), DXA-derived fat-to-lean ratio (FLR), and metabolic syndrome (MetS) were assessed. Multivariable logistic regression evaluated the association between SPISE and central adiposity. Exploratory receiver operating characteristic analyses compared the discriminatory performance of SPISE, BMI-SDS, HOMA-IR, and FLR. Results. Central adiposity was present in 58.8% of participants, whereas BMI-defined obesity occurred in 21.8%. Lower SPISE values were associated with central adiposity, obesity, MetS, HP damage, and multiple pituitary hormone deficiencies (all *p* < 0.05). SPISE declined across pubertal stages and remained associated with central adiposity after adjustment for age and selected HP variables (OR = 0.58, 95% CI 0.46–0.73). SPISE showed good discrimination of central adiposity (AUC = 0.81), with an exploratory, cohort-derived threshold of 7.82; FLR provided complementary information on body composition. Conclusions. Central adiposity was common and not fully captured by BMI-defined obesity. Lower SPISE values were associated with concurrent anthropometric and metabolic abnormalities, while DXA-derived FLR added descriptive information on body composition. Whether these indices improve cardiometabolic risk stratification beyond conventional measures requires prospective investigation.

## Simple Summary

Pediatric brain tumor survivors are at increased risk of long-term endocrine and cardiometabolic complications related to the tumor and its treatment. These alterations may include central adiposity, adverse body composition, and reduced insulin sensitivity, which may not be fully captured by body mass index alone. In this study, we evaluated the Single-Point Insulin Sensitivity Estimator (SPISE), a simple index based on routinely available clinical and laboratory parameters, together with measures of central adiposity and body composition. Lower SPISE values were associated with central adiposity and a less favorable metabolic profile, while the fat-to-lean mass ratio derived from dual-energy X-ray absorptiometry provided complementary information on the balance between fat and lean mass. These findings highlight the importance of looking beyond body mass index when assessing pediatric brain tumor survivors and support further prospective evaluation of SPISE and the fat-to-lean mass ratio as tools for metabolic phenotyping in this population.

## 1. Introduction

Over the past decades, advances in multimodal cancer therapy have substantially improved survival among children with cancer, with overall survival increasing from 56% in 1975 to more than 80% in recent years [1]. Consequently, the population of childhood cancer survivors (CCSs) has grown considerably, shifting clinical attention from survival alone to the prevention and management of long-term treatment-related complications [2]. Endocrine and metabolic disorders are among the most common late effects in CCSs, particularly among pediatric brain tumor survivors (PBTS). These complications include insulin resistance (IR), dyslipidemia, central adiposity, and metabolic syndrome (MetS), all of which may contribute to increased cardiovascular morbidity [3,4]. A universally accepted pediatric definition of MetS is still lacking, resulting in wide prevalence estimates in the general pediatric population, ranging from 0.3% to 26.4% [5]. Compared with healthy siblings, CCSs have up to a twofold higher risk of developing MetS [6], with reported prevalence estimates ranging from 6.9% to 33.6%, depending on treatment exposures and diagnostic criteria [7]. The increased metabolic burden observed in PBTSs is closely associated with tumor- and treatment-related injury to the hypothalamic–pituitary (HP) region. Surgery and cranial radiotherapy may result in growth hormone deficiency, central hypothyroidism, hypogonadism, adrenal insufficiency, and hypothalamic dysfunction, all of which are associated with obesity, adverse body composition, and cardiometabolic impairment [8,9,10,11,12,13,14]. Accordingly, current international guidelines recommend long-term endocrine and metabolic surveillance in this population [13,15].

Altered body composition, characterized by increased fat mass and reduced lean mass, is a clinically relevant feature of metabolic dysfunction in CCSs [16]. As body mass index (BMI) does not distinguish between fat and lean mass or characterize fat distribution, BMI-based assessment alone may not fully capture alterations in body composition and central adiposity. Recent findings in PBTSs further support the relevance of assessing body composition alongside conventional anthropometric measures [17]. In this context, the fat-to-lean mass ratio (FLR), derived from dual-energy X-ray absorptiometry (DXA), has been proposed as an index of relative adiposity and adverse body composition, with potential utility in CCSs [18]. Similarly, the waist-to-height ratio (WHtR) represents a simple and validated anthropometric marker of central adiposity and cardiometabolic risk in children and adolescents [19]. However, although these measures characterize adiposity and body composition, they do not directly assess the underlying metabolic abnormalities, including IR, which remain challenging to evaluate in routine pediatric practice. Although the hyperinsulinemic–euglycemic clamp is considered the gold standard for assessing insulin sensitivity, its complexity precludes routine use. Simpler surrogate indices, such as the homeostatic model assessment of insulin resistance (HOMA-IR), are limited by lack of assay standardization, pulsatile insulin secretion, and the need for pubertal stage-specific interpretation [20].

The Single-Point Insulin Sensitivity Estimator (SPISE), calculated from fasting triglycerides, high-density lipoprotein cholesterol (HDL-C), and BMI, has emerged as a practical surrogate marker associated with insulin sensitivity [21]. By integrating metabolic and anthropometric information, lower SPISE values have been associated with adverse metabolic profiles, MetS, and impaired glucose metabolism in pediatric populations with overweight or obesity [22,23,24,25]. However, evidence regarding SPISE in CCSs, particularly PBTSs, remains limited, especially concerning its association with central adiposity, DXA-derived body composition and hypothalamic–pituitary involvement.

Given the availability of SPISE components in routine clinical assessments, this study aimed to evaluate the associations of SPISE with central adiposity, metabolic abnormalities, DXA-derived body composition, and tumor- and treatment-related factors affecting the hypothalamic–pituitary (HP) region in PBTSs. We also explored whether DXA-derived FLR could provide complementary information on body composition and metabolic vulnerability in this population.

## 2. Materials and Methods

### 2.1. Study Design and Population

This retrospective cross-sectional study included brain tumor survivors aged 5–18 years who were evaluated between July 2008 and May 2025 at the Pediatric Endocrinology Unit of the IRCCS Giannina Gaslini Institute, Genoa, Italy. Eligibility criteria included completion of cancer treatment at least two years before the study evaluation and the absence of acute intercurrent illness or ongoing pharmacological treatments known to substantially affect glucose metabolism, except for stable replacement therapy for documented pituitary hormone deficiencies.

The study was reported in accordance with the Strengthening the Reporting of Observational Studies in Epidemiology (STROBE) guidelines for cross-sectional studies.

### 2.2. Data Collection

This study was approved by the National Ethics Committee for Clinical Trials (protocol MOMANADI_001_rev02; registration number 429REG2017). Written informed consent was obtained from parents or legal guardians, and assent was obtained from participants aged ≥5 years, as appropriate. Participants underwent a single study evaluation that included a clinical assessment, standardized anthropometric measurements, DXA-derived body composition assessment, and fasting biochemical testing. Demographic, oncological, endocrine, auxological, biochemical, and neuroradiological data were retrospectively extracted from medical records.

#### 2.2.1. Oncological and Endocrine History

Tumors were classified according to the 2016 World Health Organization classification of brain tumors [26]. Treatment data included surgery, chemotherapy, and radiotherapy (RT), with craniospinal RT recorded separately when applicable.

HP tumor involvement was defined on the basis of neuroradiological evidence of tumor infiltration, compression, or displacement of the hypothalamus and/or pituitary stalk. Available magnetic resonance images were reviewed by an experienced neuroradiologist (A.R.). Pituitary dysfunction was assessed separately from anatomical tumor involvement.

Treatment involving the HP region was defined as neurosurgery directly involving the sellar or suprasellar region and/or radiotherapy encompassing the HP region.

The presence of hydrocephalus and the number and type of post-treatment pituitary hormone deficiencies were recorded, including growth hormone deficiency (GHD), thyroid-stimulating hormone deficiency (TSHD), adrenocorticotropic hormone deficiency (ACTHD), gonadotropin deficiency (GnD), and arginine vasopressin deficiency (AVPD).

#### 2.2.2. Auxological and Pubertal Assessment

Height and weight were obtained during standardized anthropometric assessments. Body mass index (BMI) was calculated as weight in kilograms divided by height in meters squared and expressed as a standard deviation score (BMI-SDS) according to World Health Organization reference standards [27]. Overweight and obesity were defined as BMI-SDS ≥ +1 and BMI-SDS ≥ +2, respectively.

Waist circumference (WC) and hip circumference (HC) were measured using a non-elastic tape according to standardized procedures. These measurements were used to calculate the waist-to-hip ratio and WHtR [19]. A WHtR ≥ 0.5 was considered indicative of central adiposity and used as a pragmatic marker associated with increased cardiometabolic risk [28].

Systolic and diastolic blood pressure (SBP and DBP) were measured during the clinical evaluation. Pubertal development was assessed according to Tanner staging and categorized as prepubertal (Tanner stage 1), pubertal (Tanner stages 2–4), or postpubertal (Tanner stage 5).

#### 2.2.3. Body Composition

Total-body-less-head (TBLH) DXA scans were performed to assess body composition. Depending on the study period, measurements were obtained using GE Lunar Prodigy systems (software version 16) or iDXA systems (software version 18). Both systems were manufactured by GE Healthcare and were operated according to standardized manufacturer procedures.

TBLH fat mass (FM) and trunk FM were expressed in kilograms (kg) and as a percentage (%), whereas TBLH lean mass (LM) was expressed in kilograms (kg). The FLR was calculated as TBLH FM divided by TBLH LM and was used as an index of relative adiposity and body composition.

#### 2.2.4. Biochemical Parameters

Fasting blood samples obtained during routine follow-up evaluations were analyzed in the same institutional laboratory. Recorded parameters included fasting glucose, insulin, glycated hemoglobin (HbA1c), triglycerides, total cholesterol, high-density lipoprotein cholesterol (HDL-C), low-density lipoprotein cholesterol (LDL-C), alanine aminotransferase (ALT), and aspartate aminotransferase (AST).

Insulin sensitivity was retrospectively estimated using the following indices:

– SPISE = 600 × HDL-C^0.185^/(TG^0.2^ × BMI^1.338^), where TG and HDL-C are expressed in mg/dL and BMI in kg/m^2^ [21].
– HOMA-IR = [Fasting insulin (μU/mL) × fasting glucose (mmol/L)]/22.5 [20].

Lower SPISE values indicate lower estimated insulin sensitivity, whereas higher HOMA-IR values indicate greater estimated insulin resistance.

#### 2.2.5. Metabolic Syndrome

Metabolic syndrome (MetS) was assessed according to the International Diabetes Federation (IDF) consensus criteria for children and adolescents [29]. In participants aged <10 years, MetS was not formally diagnosed; however, central obesity, defined as WC ≥ 90th percentile for age and sex, and the individual metabolic abnormalities were recorded.

In participants aged ≥10 years, MetS was diagnosed in the presence of central obesity plus at least two additional metabolic abnormalities. Central obesity was defined as WC ≥ 90th percentile for age and sex in participants aged 10 to <16 years, using the international reference values proposed by Xi et al. [30], and as WC ≥ 94 cm in males and ≥80 cm in females aged ≥16 years. The additional metabolic abnormalities and their corresponding diagnostic criteria are summarized in Table 1.

### 2.3. Statistical Analysis

Central adiposity, defined as WHtR ≥ 0.5, was considered the primary outcome and was used as a pragmatic marker associated with increased cardiometabolic risk. Secondary outcomes included MetS, the DXA-derived FLR, and the associations of SPISE and FLR with anthropometric, metabolic, oncological, and endocrine variables.

Descriptive statistics were used to characterize the study population. Normality was assessed using the Shapiro–Wilk test together with visual inspection of histograms and Q–Q plots. Because most continuous variables showed non-normal distributions, non-parametric tests were used. Continuous variables are presented as median and interquartile range [25th, 75th percentile] and categorical variables as numbers and percentages.

Participants were stratified according to BMI-SDS (≥2 vs. <2) and WHtR (≥0.5 vs. <0.5). Continuous variables were compared using the Mann–Whitney U test for two-group comparisons or the Kruskal–Wallis test for comparisons involving more than two groups, as appropriate. Categorical variables were compared using the chi-square test or Fisher’s exact test, as appropriate.

The association between SPISE and central adiposity was assessed using logistic regression, with results expressed as odds ratios (ORs) and 95% confidence intervals (CIs). Multivariable logistic regression models were constructed to evaluate the association between SPISE and central adiposity, adjusting for a limited number of covariates selected on the basis of clinical relevance, biological plausibility and univariable findings, while considering sample size, collinearity, and the risk of model overfitting.

Exploratory receiver operating characteristic (ROC) curve analyses were performed to assess the discriminatory performance of SPISE, BMI-SDS, HOMA-IR, and FLR for central adiposity. The area under the curve (AUC), 95% CI, sensitivity, specificity, and optimal cohort-derived cutoff values based on the Youden index were calculated. The AUCs of SPISE, BMI-SDS, and HOMA-IR were compared using DeLong’s test.

Only participants with complete data for the variables included in each analysis were considered; no data imputation was performed. Outliers were assessed by graphical inspection and verified against the source data. All statistical analyses were performed using SPSS Statistics, version 22.0 (IBM Corp., Armonk, NY, USA). A two-sided *p* value < 0.05 was considered statistically significant.

## 3. Results

### 3.1. Study Cohort

Of the 132 PBTSs screened, 119 had complete data for calculation of WHtR, SPISE, and DXA-derived FLR and were included. The cohort included 58 males (48.7%) and had a median age at evaluation of 12.7 years [9.5, 14.9]. The median age at diagnosis was 7.1 years [3.8, 10.0] and the interval since completion of cancer therapy was 2.4 years [2.1, 5.1]. Overall, 35 participants (29.4%) were prepubertal, 66 (55.5%) were at Tanner stages 2–4, and 18 (15.1%) were postpubertal. Age increased significantly across Tanner stages (*p* < 0.001).

Clinical, oncological, and endocrine characteristics are summarized in Table 2. Glial tumors (32.8%) and embryonal tumors (26.9%) were the most frequent diagnoses, whereas craniopharyngiomas, germ cell tumors, and ependymomas each accounted for approximately 10–14% of cases. Overall, 85 participants (71.4%) underwent surgery and/or radiotherapy involving the hypothalamic–pituitary region. Regarding pituitary function, 23 participants (19.3%) had no pituitary hormone deficiencies, 61 (51.3%) had one or two deficiencies, and 35 (29.4%) had three or more deficiencies.

### 3.2. Anthropometric and Body Composition Profile

Obesity, defined according to BMI criteria, was present in 21.8% of participants and was similarly distributed between males and females. Female participants had a higher TBLH FM percentage, lower TBLH LM, and higher FLR than males (all *p* < 0.01; Table 3).

**Figure 1:**
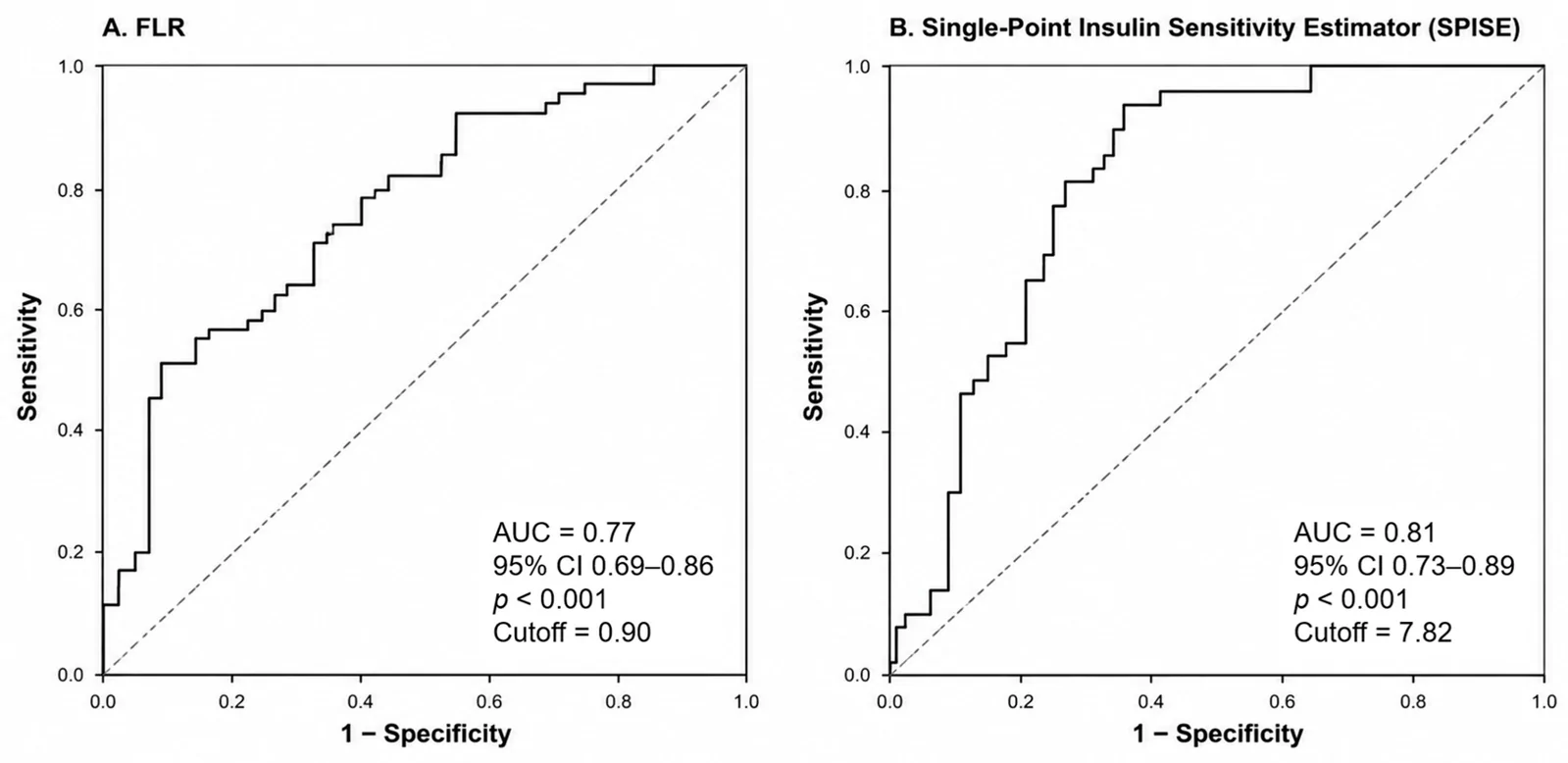
Receiver operating characteristic (ROC) curves showing the performance of (**A**) the fat-to-lean mass ratio (FLR) and (**B**) the Single-Point Insulin Sensitivity Estimator (SPISE) in discriminating participants with central adiposity (defined as WHtR ≥ 0.5). Note: In these exploratory analyses, the area under the curve (AUC) was 0.77 (95% CI 0.69–0.86; *p* < 0.001) for FLR and 0.81 (95% CI 0.73–0.89; *p* < 0.001) for SPISE. The cohort-derived cutoffs were 0.90 for FLR (sensitivity 51%, specificity 92%) and 7.82 for SPISE (sensitivity 64%, specificity 94%). The diagonal line represents discrimination equivalent to chance (AUC = 0.50).

Central adiposity, defined as WHtR ≥ 0.5, was present in 58.8% of the cohort, with a similar prevalence in males and females. Participants with WHtR-defined central adiposity had a less favorable body composition, with higher total and trunk FM (in kg and as a percentage) and a higher FLR (all *p* < 0.001; Table 4), but no difference in LM. In contrast, patients with BMI-defined obesity presented an increase in both fat and lean mass (Table 4). Exploratory ROC curve analysis identified a FLR threshold of ≥0.90 for discriminating participants with central adiposity (AUC = 0.77, 95% CI 0.69–0.86; *p* < 0.001), with 51% sensitivity and 92% specificity (Figure 1A).

Both WHtR and FLR differed across tumor diagnostic groups (overall *p* = 0.003 and *p* = 0.017, respectively), with the highest values observed among survivors of glial tumors and choroid plexus tumors. WHtR also differed according to the burden of pituitary hormone deficiencies (*p* = 0.020), with higher values in participants with more deficiencies. FLR was not significantly associated with the number of pituitary hormone deficiencies or pubertal stage (*p* = 0.282 and *p* = 0.614, respectively).

### 3.3. SPISE and Cardiometabolic Profiles

No significant sex-related differences were observed in SPISE values (Table 3). In contrast, SPISE differed significantly across pubertal stages (*p* = 0.010), with a progressive decline from prepubertal to postpubertal stages: 9.83 [7.50, 13.29] at Tanner stage 1, 8.39 [6.83, 10.24] at Tanner stages 2–4, and 6.89 [4.88, 9.73] at Tanner stage 5.

Lower SPISE values were observed in participants with central adiposity than in those without central adiposity (7.08 [5.06, 8.97] vs. 10.09 [8.78, 12.16]; *p* < 0.001; Table 4). Similarly, participants with BMI-SDS ≥ +2 had lower SPISE values than those with BMI-SDS < +2 (4.86 [4.14, 6.83] vs. 9.14 [7.72, 10.85]; *p* < 0.001). Participants with overweight, defined as BMI-SDS ≥ +1, also had lower SPISE values than those with BMI-SDS < +1 (6.84 [4.90, 7.98] vs. 10.42 [8.78, 12.46]; *p* < 0.001).

### 3.4. SPISE in Relation to Oncologic and Endocrine Factors

**Figure 2:**
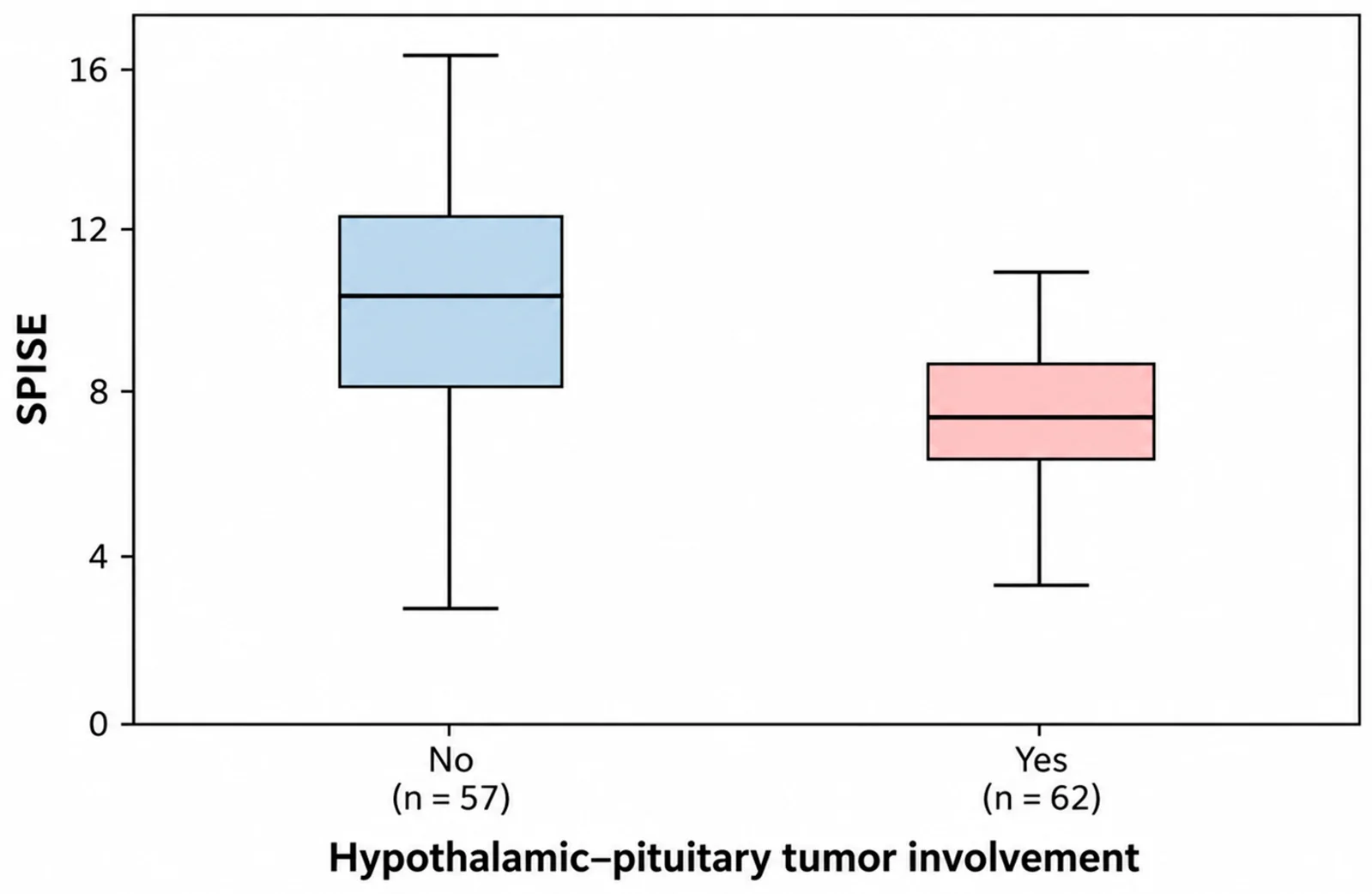
Distribution of SPISE values according to hypothalamic–pituitary involvement of the HP region. SPISE values were lower in participants with HP tumor involvement (n = 62; median 7.24 [6.30, 8.99]) than in those without involvement (n = 57; median 9.93 [7.92, 12.01]; *p* < 0.001). Note: Box plots represent the median (horizontal line), interquartile range (box edges 25th, 75th percentiles), and non-outlier range (whiskers).

SPISE values differed significantly across tumor types (*p* = 0.001), with lower values among survivors of glial tumors, craniopharyngiomas, and choroid plexus tumors (Table 5). When treatments involving the HP region were considered separately, SPISE values differed significantly across groups (*p* = 0.002), with the lowest values in participants exposed to both surgery and radiotherapy involving this region. Similarly, survivors with tumoral involvement of the HP region had lower SPISE values than those without such involvement (*p* < 0.001; Figure 2). SPISE also differed according to endocrine burden, with the lowest values observed among survivors with three or more pituitary hormone deficiencies (*p* = 0.013). In contrast, SPISE was not significantly associated with hydrocephalus at tumoral diagnosis.

### 3.5. Associations and Discriminative Performance of SPISE for Central Adiposity

**Figure 3:**
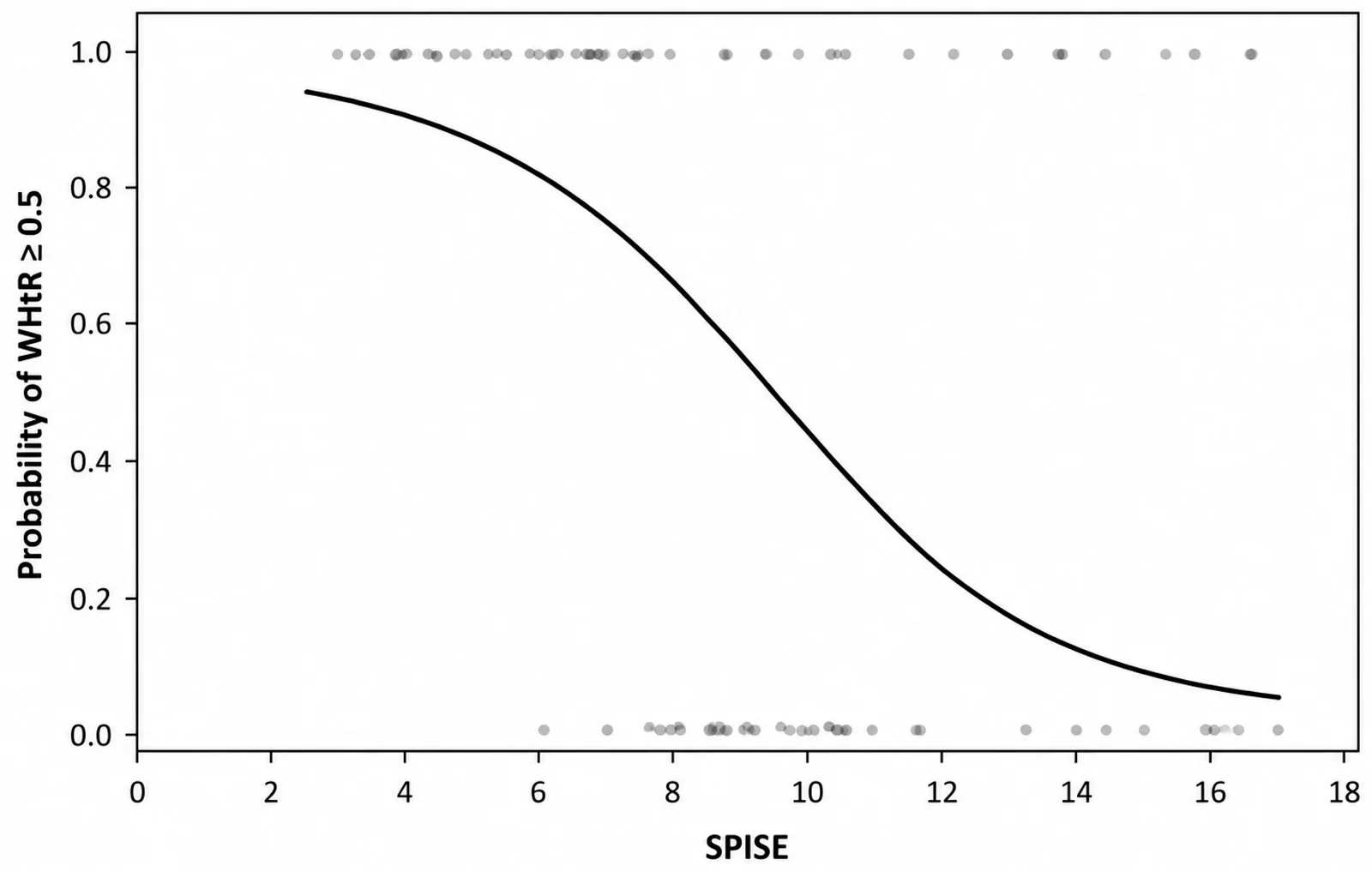
Association between SPISE and central adiposity, defined as WHtR ≥ 0.5, in pediatric brain tumor survivors. The solid line represents the estimated probability of central adiposity across SPISE values based on a univariable logistic regression model (OR = 0.69 per 1-unit increase in SPISE; 95% CI 0.58–0.81; *p* < 0.001). Gray dots represent individual observations (0 = WHtR < 0.5; 1 = WHtR ≥ 0.5), with vertical jitter applied to reduce overlap.

Logistic regression analysis showed a significant inverse association between SPISE and central adiposity, defined as WHtR ≥ 0.5 (OR = 0.69 per 1-unit increase in SPISE, 95% CI 0.58–0.81; *p* < 0.001; Figure 3). In univariate analysis, central adiposity was not significantly associated with sex (*p* = 0.687), Tanner stage (*p* = 0.352) or time off therapy (*p* = 0.238). Consequently, these variables were not included in the multivariable models.

**Figure 4:**
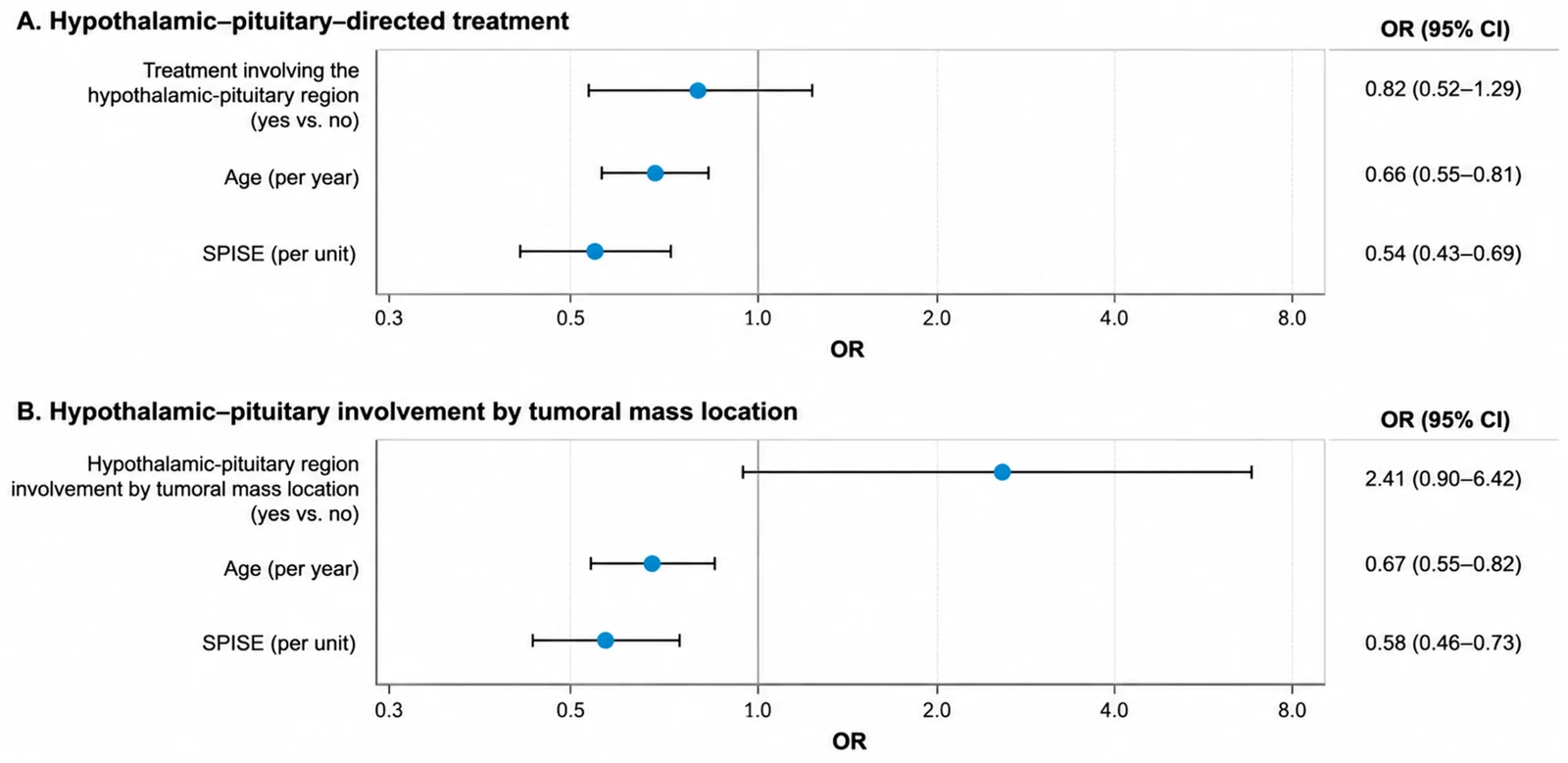
Multivariable logistic regression models for central adiposity, defined as WHtR ≥ 0.5, in pediatric brain tumor survivors. (**A**) Model including SPISE, age at evaluation, and treatment involving the HP region. (**B**) Model including SPISE, age at evaluation, and tumoral involvement of the HP region. Forest plots show odds ratios (ORs) and 95% confidence intervals (CIs) on a logarithmic (log_2_) scale, with numerical estimates displayed alongside each variable. The vertical reference line at OR = 1 indicates no association. ORs for SPISE and age refer to a 1-unit increase and a 1-year increase, respectively. For treatment involving the HP region, “no treatment” is the reference category (yes vs. no); for tumoral involvement of the HP region, “no involvement” is the reference category (yes vs. no).

In the multivariable logistic regression models, lower SPISE values and younger age were associated with central adiposity after adjustment for the other covariates included in each model. In Model 1 (Figure 4A), SPISE (OR = 0.54 per 1-unit increase, 95% CI 0.43–0.69; *p* < 0.001) and age (OR = 0.66 per 1-year increase, 95% CI 0.55–0.81; *p* < 0.001) were associated with the outcome, whereas treatment involving the hypothalamic–pituitary region was not (OR = 0.82, 95% CI 0.52–1.29; *p* = 0.402). In Model 2 (Figure 4B), SPISE (OR = 0.58 per 1-unit increase, 95% CI 0.46–0.73; *p* < 0.001) and age (OR = 0.67 per 1-year increase, 95% CI 0.55–0.82; *p* < 0.001) remained associated with central adiposity, whereas tumor involvement of the HP region showed a trend toward significance (OR = 2.41, 95% CI 0.90–6.42; *p* = 0.079).

To further account for age at diagnosis, this variable was incorporated into Models 3 and 4. In Model 3, age at diagnosis (OR = 0.75, 95% CI 0.64–0.87, *p* < 0.001) and SPISE (OR = 0.59, 95% CI 0.48–0.73, *p* < 0.001) were associated with central adiposity, whereas treatment involving the HP region was not (OR = 0.94, 95% CI 0.61–1.46, *p* = 0.795). In Model 4, age at diagnosis (OR = 0.73, 95% CI 0.62–0.86, *p* < 0.001), SPISE (OR = 0.63, 95% CI 0.52–0.77, *p* < 0.001) and tumoral involvement of the HP region (OR = 3.24, 95% CI 1.18–8.89, *p* = 0.022) were associated with central adiposity.

Exploratory ROC curve analysis yielded an AUC of 0.81 (95% CI 0.73–0.89; *p* < 0.001) and identified a cohort-derived SPISE threshold of 7.82 for discriminating participants with central adiposity, with 64% sensitivity and 94% specificity (Figure 1B). Participants with SPISE ≤ 7.82 had higher WHtR, greater FM, higher FLR and HOMA-IR, and higher blood pressure than those with SPISE > 7.82 (Table 6). As expected from the mathematical definition of SPISE, BMI, triglycerides, and HDL-C also differed between groups.

In exploratory ROC analyses, SPISE and BMI-SDS showed comparable discriminatory performance for central adiposity, with AUCs of 0.81 (95% CI 0.73–0.89) and 0.85 (95% CI 0.78–0.92), respectively (DeLong *p* = 0.41). HOMA-IR showed lower discriminatory performance than SPISE (AUC = 0.63, 95% CI 0.53–0.73; DeLong *p* = 0.008).

### 3.6. Metabolic Syndrome and Its Components

Among the 119 participants, 33 (27.7%) were aged <10 years and were therefore not formally evaluable for MetS according to IDF criteria, whereas 86 (72.3%) were aged ≥10 years and were eligible for MetS assessment.

Among participants aged <10 years, central obesity, defined as WC ≥ 90th percentile for age and sex, was present in 15 of 33 children (45.5%).

Among the 86 participants aged ≥10 years, nine met the criteria for MetS, corresponding to an overall prevalence of 10.5% (six males and three females). By tumor histology, MetS was observed in four participants with astrocytomas, three with craniopharyngiomas, and two with other glial tumors.

Among participants aged 10 to <16 years (n = 70), central obesity was present in 22 (31.4%), hypertriglyceridemia in eight (11.4%), low HDL-C in 11 (15.7%), impaired fasting glucose in one (1.4%), and arterial hypertension in one (1.4%). Overall, five participants in this age group (7.1%) met the criteria for MetS.

Among participants aged ≥16 years (n = 16), central obesity, defined as WC ≥ 94 cm in males and ≥80 cm in females, was present in eight (50.0%), hypertriglyceridemia in five (31.3%), low HDL-C in eight (50.0%), impaired fasting glucose in two (12.5%), and arterial hypertension in one (6.3%). Overall, four participants in this age group (25.0%) met the criteria for MetS.

Participants with MetS had significantly lower SPISE values than those without MetS (4.08 [3.46, 5.69] vs. 8.40 [6.89, 10.16]; *p* < 0.001).

## 4. Discussion

CCSs are at increased risk of endocrine and metabolic sequelae, with a substantial proportion developing chronic health conditions during long-term follow-up [13,31]. Data from the Childhood Cancer Survivor Study indicate that the incidence of diabetes is nearly twice that in the general population [32]. In PBTSs, tumor- and treatment-related injury to the HP region may contribute to pituitary hormone deficiencies, central adiposity, adverse body composition, and insulin resistance [33]. Because these abnormalities may precede overt dysglycemia and MetS, their early recognition is relevant to long-term cardiometabolic surveillance [34]. However, direct methods for assessing insulin sensitivity are invasive and complex, while available surrogate indices remain insufficiently standardized in children and adolescents [20]. In this context, SPISE has emerged as a practical surrogate index of insulin sensitivity, calculated from routinely available anthropometric and fasting biochemical measurements [21].

To our knowledge, this is the first study to evaluate SPISE in a pediatric cohort of brain tumor survivors, examining its associations with central adiposity, body composition, metabolic characteristics, and HP involvement. Although only approximately one-fifth of participants had BMI-defined obesity, more than half had central adiposity. This discrepancy highlights the limitations of BMI alone in characterizing the adiposity and body composition phenotype of this population. A cohort of 21 childhood brain tumor survivors reported a similar prevalence of central adiposity (WHtR > 0.5 in 68%); however, participants in that cohort were older at evaluation (mean age, 22.4 years) than those in our study [35]. More recently, a study involving 30 childhood brain tumor survivors reported that 53% were classified as normal weight, whereas 59% of those assessed for body composition (n = 29) had an elevated body fat percentage, further highlighting the limitations of BMI in characterizing adiposity in this population [17]. Together, these findings support the relevance of assessing central adiposity and body composition alongside BMI during survivorship follow-up. Lower SPISE values were observed in participants with central adiposity, BMI-defined obesity, and MetS. SPISE also remained associated with central adiposity after adjustment for age at evaluation or age at brain tumor diagnosis and selected HP variables. These findings support an association between SPISE and concurrent anthropometric and metabolic abnormalities in PBTSs but do not establish additional clinical utility beyond conventional measures of adiposity.

SPISE values also differed across tumor and treatment subgroups. Numerically lower values were observed in survivors of craniopharyngiomas and glial tumors, as well as among participants with tumoral involvement of the HP region, exposure to surgery and/or radiotherapy involving this region, and a greater burden of pituitary hormone deficiencies. These findings are consistent with the contribution of HP injury and endocrine dysfunction to the adverse metabolic phenotype observed in brain tumor survivors [10,12,36]. A recent retrospective study of 388 childhood brain tumor patients reported endocrine disorders in 75.8% of the cohort, with the highest prevalence among patients with craniopharyngiomas (90.0%) and germ-cell tumors (92.2%). Sellar/suprasellar tumor location and younger age at diagnosis were identified as risk factors for endocrine morbidity [37]. Although metabolic outcomes were not assessed in that study, these findings further highlight the importance of considering tumor-related and endocrine factors during long-term follow-up. Current survivorship recommendations include regular assessment of BMI, blood pressure, and metabolic parameters, including fasting lipid profiles in at-risk survivors [4]. However, BMI may underestimate adiposity and related metabolic risk in this population. SPISE is a readily calculated surrogate index that integrates anthropometric and lipid parameters collected during routine cardiometabolic follow-up. Whether it provides clinically relevant information beyond these individual measurements remains to be established.

Across the multivariable models, lower SPISE values remained associated with central adiposity after adjustment for selected age-related and HP variables. Tumoral involvement of the HP region showed a positive but imprecisely estimated association with central adiposity in Model 2 (OR approximately 2.4) and was statistically significant in Model 4 after adjustment for age at diagnosis (OR approximately 3.2). In contrast, treatment involving the HP region was not significantly associated with central adiposity in either model. These findings should be interpreted cautiously, given the limited sample size, potential residual confounding, and differences in covariate adjustment. Although hypothalamic dysfunction may contribute to the observed metabolic phenotype, the present analyses cannot establish the underlying mechanisms.

Physiological maturation is important when interpreting SPISE in pediatric populations. SPISE declined across pubertal stages, consistent with the physiological reduction in insulin sensitivity that occurs during puberty. Unexpectedly, older age was associated with lower odds of central adiposity, consistent with the younger age observed among participants with WHtR ≥ 0.5. This association should be interpreted cautiously and not as a protective effect of increasing age. Chronological age may reflect differences in pubertal maturation, age at tumor diagnosis, treatment exposures, and time since treatment, which cannot be fully disentangled in this heterogeneous cohort. The association between lower SPISE and central adiposity persisted after adjustment for age, suggesting that it was not explained by chronological age alone. Nevertheless, the cross-sectional design does not allow age to be used as a proxy for the duration of central adiposity or to distinguish early-onset, persistent adiposity from more recent onset.

Unlike SPISE, DXA-derived FLR did not differ significantly across pubertal stages. However, this finding does not exclude an influence of maturation on body composition. By expressing FM relative to LM, FLR provides information on disproportionate adiposity that is not captured by BMI or WHtR. In a recent study of 21 pediatric craniopharyngioma patients, bioelectrical impedance analysis (BIA) showed higher total and relative trunk adiposity and a lower muscle-to-fat ratio compared with age- and sex-matched controls, despite no significant difference in appendicular skeletal muscle mass z-scores [38]. Although BIA and DXA use different methodologies and the indices examined are not directly interchangeable, these findings are consistent with the importance of characterizing fat and lean compartments beyond conventional anthropometric measures. In our cohort, female participants had a higher FM percentage and FLR and lower LM than males; these differences may reflect physiological sex-related variation rather than a more adverse body composition phenotype. Without age-, sex-, and maturation-appropriate reference values, the clinical significance of these differences remains uncertain. Moreover, FLR should not be interpreted as a direct measure of sarcopenia because muscle strength and physical performance were not assessed.

The findings also illustrate the mathematical and biological differences among SPISE, BMI-SDS, HOMA-IR, WHtR, and FLR. In exploratory ROC analyses, SPISE showed greater discrimination of central adiposity than HOMA-IR, but discrimination comparable to that of BMI-SDS. This comparison should be interpreted cautiously. HOMA-IR reflects fasting glucose–insulin homeostasis, whereas SPISE incorporates BMI, triglycerides, and HDL-C. Because BMI is mathematically embedded in SPISE and is closely related to WHtR, some of the observed discriminatory performance of SPISE may be attributable to this intrinsic anthropometric overlap. Importantly, these findings do not demonstrate an incremental discriminatory benefit of SPISE over BMI-SDS for identifying central adiposity. Although SPISE incorporates lipid variables in addition to BMI, its ability to discriminate WHtR-defined central adiposity does not establish added value over anthropometry. Whether SPISE improves cardiometabolic risk stratification beyond conventional measures requires further investigation.

The exploratory SPISE threshold identified in this cohort, 7.82, was higher than values reported in non-oncological pediatric populations. Previous studies identified a threshold of 6.08 associated with abnormal glucose metabolism in children and adolescents with overweight or obesity [24], whereas Korean population-based studies proposed thresholds of 6.5 for MetS [25] and 7.0 for pediatric metabolic dysfunction-associated steatotic liver disease [39]. Differences among studies may reflect variation in age, pubertal status, BMI distribution, ethnicity, outcome definition, and clinical setting rather than a threshold specific to PBTSs. Accordingly, the proposed SPISE threshold should be considered cohort-derived and exploratory until externally validated.

Similarly, the FLR threshold of 0.90 identified in this cohort was higher than the previously reported value of 0.60 in childhood cancer survivors [18]. This difference may reflect variation in cohort composition, sex distribution, pubertal status, tumor location, treatment exposure, endocrine dysfunction, and DXA methodology. In the present cohort, both FLR ≥ 0.90 and SPISE ≤ 7.82 showed high specificity for central adiposity, although sensitivity values differed. These findings suggest that SPISE and FLR provide different descriptive information on metabolic and body composition profiles, but do not demonstrate that their combined use improves cardiometabolic risk discrimination. Both thresholds were derived and evaluated in the same cohort and should not be used for clinical decision-making without independent validation.

The prevalence of MetS among participants aged ≥10 years was 10.5%, within the broad range reported in pediatric cancer survivors, although estimates vary considerably according to age, treatment exposure, and diagnostic criteria [5,6]. Participants with MetS had markedly lower SPISE values than those without MetS. This association is biologically plausible but should be interpreted cautiously because triglycerides and HDL-C are components of both SPISE and the MetS definition. Therefore, the association does not represent independent validation of SPISE as a marker of MetS.

Overall, these findings support the need for careful metabolic surveillance in PBTSs and are consistent with current recommendations from the International Late Effects of Childhood Cancer Guideline Harmonization Group, which emphasize the assessment of central adiposity, blood pressure, glucose metabolism, and lipid abnormalities during long-term follow-up [15]. Participants with central adiposity, BMI-defined obesity, low SPISE, and MetS showed differences in concurrent metabolic profiles, with more pronounced abnormalities observed in some subgroups. However, these categories are not mutually exclusive, and their overlap, together with their cross-sectional design, precludes interpreting these findings as a progression from early central adiposity to more advanced cardiometabolic impairment. Prospective longitudinal studies incorporating direct measures of insulin sensitivity and clinically relevant outcomes are needed to determine whether SPISE and DXA-derived FLR improve risk stratification beyond conventional anthropometric and biochemical assessment.

The main strength of this study is the comprehensive characterization of a clinically relevant cohort of PBTSs, integrating oncological history, hypothalamic–pituitary involvement, endocrine status, pubertal stage, anthropometric measures, biochemical variables, and DXA-derived body composition. To our knowledge, this is the first study to evaluate SPISE in relation to central adiposity, body composition, and hypothalamic–pituitary tumor- and treatment-related factors in this population.

Several limitations should be acknowledged. First, the retrospective cross-sectional design precludes conclusions regarding temporality, causality, or prediction of future cardiometabolic outcomes. Second, the single-center setting and modest sample size may limit generalizability and the precision of multivariable estimates, particularly in smaller tumor- and treatment-specific subgroups. Although sex and pubertal stage were evaluated in univariable analyses, they were not included in the multivariable models. Adjustment for chronological age may not fully account for pubertal maturation, particularly in survivors with hypothalamic–pituitary dysfunction, and residual confounding cannot be excluded. Third, detailed adjustment for hormone replacement therapies, radiotherapy doses, and other treatment-related factors was not feasible, leaving the possibility of residual confounding. Moreover, the high prevalence of radiotherapy exposure and the heterogeneity of tumor locations and treatment regimens limit our ability to assess potential metabolic differences between irradiated and non-irradiated survivors. A further limitation concerns the mathematical structure of SPISE. Because the index incorporates BMI, triglycerides, and HDL-C, its associations with WHtR, BMI-SDS, and MetS may partly reflect shared or correlated components rather than independent metabolic information. Although WHtR is not directly included in the SPISE equation, its association with BMI may contribute to the observed relationship. Furthermore, triglycerides and HDL-C are components of both SPISE and the MetS definition, limiting the independent interpretation of this association. Accordingly, comparisons of BMI, triglycerides, and HDL-C across SPISE-defined groups should be considered descriptive and should not be interpreted as independent validation of SPISE. In addition, the absence of a direct measure of insulin sensitivity limits the physiological interpretation of SPISE. Finally, both the SPISE and FLR thresholds were derived and evaluated within the same cohort, which may have resulted in optimistic estimates of discriminatory performance. These thresholds should therefore be considered exploratory until externally validated in independent longitudinal cohorts. FLR was not interpreted against age-, sex-, and maturation-specific reference values, limiting the clinical interpretation of observed differences in body composition.

## 5. Conclusions

PBTSs showed a high prevalence of central adiposity that was not fully captured by BMI-defined obesity. Lower SPISE values were associated with central adiposity and a less favorable metabolic profile, with differences also observed according to HP involvement and the burden of hormone deficiencies, while DXA-derived FLR provided complementary information on body composition. These findings highlight the importance of assessing central adiposity and metabolic characteristics alongside BMI during long-term follow-up. Further prospective studies are needed to determine whether SPISE and FLR offer incremental clinical utility beyond conventional anthropometric and biochemical measures for cardiometabolic risk assessment in this population.

## Figures and Tables

**Table 1:** Additional metabolic abnormalities included in the IDF criteria for metabolic syndrome.

Metabolic Abnormality	Diagnostic Criteria
Elevated triglycerides	Triglycerides ≥ 150 mg/dL or specific treatment for hypertriglyceridemia
Reduced HDL-C	Age 10 to <16 years: HDL_C < 40 mg/dL Age ≥ 16 years: HDL-C < 40 mg/dL in males and <50 mg/dL in females, or specific treatment for low HDL-C
Elevated blood pressure	SBP ≥ 130 mmHg or DBP ≥ 85 mmHg, or treatment for previously diagnosed hypertension
Elevated fasting glucose	Fasting plasma glucose ≥ 100 mg/dL or previously diagnosed type 2 diabetes

Abbreviations: IDF, International Diabetes Federation; HDL-C, high-density lipoprotein cholesterol; SBP, systolic blood pressure; DBP, diastolic blood pressure.

**Table 2:** Clinical, oncological, and endocrine characteristics of the study cohort.

	n = 119
Demographic characteristics	
Gender, n (%)	
Male	58 (48.7%)
Female	61 (51.3%)
Age (years), median [25th, 75th percentile]	
At diagnosis	7.1 [3.8, 10.0]
At the end of therapies	8.7 [5.8, 11.5]
At follow-up	12.7 [9.5, 14.9]
Time since off therapy (years), median [25th, 75th percentile]	2.4 [2.1, 5.1]
Tanner stage at follow-up, n (%)	
Tanner 1	35 (29.4%)
Tanner 2 to 4	66 (55.5%)
Tanner 5	18 (15.1%)
Tumor characteristics and treatment	
Diagnosis, n (%)	
Glial tumors	39 (32.8%)
Embryonal tumors	32 (26.9%)
Craniopharyngioma	17 (14.3%)
Germ cell tumors	16 (13.4%)
Ependymoma	12 (10.1%)
Choroid plexus tumors	2 (1.7%)
GH-secreting pituitary adenoma	1 (0.8%)
Anticancer treatments, n (%)	
Radiotherapy	99 (83.2%)
Craniospinal radiotherapy	37 (31.1%)
Chemotherapy	86 (72.3%)
Surgery	96 (80.7%)
Treatments involving the hypothalamic–pituitary region, n (%)	
None	34 (28.6%)
Surgery only	8 (6.7%)
Radiotherapy only	51 (42.9%)
Surgery + radiotherapy	26 (21.8%)
Tumoral mass involving the hypothalamic–pituitary region, n (%)	
Yes	62 (52.1%)
No	57 (47.9%)
Presence of hydrocephalus, n (%)	
Yes	63 (52.9%)
No	56 (47.1%)
Endocrine outcomes	
Post-treatment hormone deficiencies, n (%)	
GHD	71 (59.7%)
TSHD	53 (44.5%)
ACTHD	40 (33.6%)
GnD assessed in participants aged >13 years for females and >14 years for males (n = 60)	23 (38.3%)
Hypogonadotropic hypogonadism	19 (82.6%)
Hypergonadotropic hypogonadism	4 (17.4%)
AVPD	27 (22.7%)
Number of post-treatment pituitary hormone deficiencies, n (%)	
0	23 (19.3%)
1	34 (28.6%)
2	27 (22.7%)
3	11 (9.2%)
4	14 (11.8%)
5	10 (8.4%)
Metabolic syndrome	
Presence of metabolic syndrome, n (%)	
Overall (age ≥ 10 years, n = 86)	9 (10.5%)
Age 10 to <16 years (n = 70)	5 (7.1%)
Age ≥ 16 years (n = 16)	4 (25.0%)

Abbreviations: GHD, growth hormone deficiency; TSHD, thyroid-stimulating hormone deficiency; ACTHD, adrenocorticotropic hormone deficiency; GnD, gonadotropin deficiency; AVPD, arginine vasopressin deficiency. Note: Glial tumors include astrocytoma, ganglioglioma, glioma (including neurofibromatosis type 1 (NF1)-associated forms), gliomatosis, and glioblastoma. Embryonal tumors include medulloblastoma, primitive neuroectodermal tumor (PNET), atypical teratoid/rhabdoid tumor (AT/RT), malignant embryonal tumor, and ganglioneuroblastoma. Germ cell tumors include germinoma, teratoma, and mixed germinoma/non-germinomatous tumors. GH-secreting pituitary adenoma, choroid plexus tumors, craniopharyngioma, and ependymoma are reported as separate diagnostic categories. Anticancer treatment categories are not mutually exclusive. Percentages for GHD, TSHD, ACTHD, and AVPD were calculated using the entire cohort (n = 119). GnD was assessed in participants aged >13 years for females and >14 years for males (n = 60). Percentages for hypogonadotropic and hypergonadotropic hypogonadism were calculated among participants with GnD (n = 23).

**Table 3:** Anthropometric, body composition, metabolic, and blood pressure characteristics according to sex.

	All (n = 119)	Male (n = 58)	Female (n = 61)	*p* Value
Demographic, anthropometric and pubertal characteristics				
Age at diagnosis (years)	7.1 [3.8, 10.0]	7.2 [3.9, 10.8]	6.3 [3.5, 9.6]	0.243
Age at follow-up (years)	12.7 [9.5, 14.9]	13.2 [9.9, 15.4]	12.3 [8.9, 14.5]	0.177
Height SDS	−0.40 [−1.60, 0.60]	−0.35 [−1.45, 0.87]	−0.80 [−1.90, 0.10]	0.071
Tanner 1	35 (29.4%)	20 (34.5%)	15 (24.6%)	0.447
Tanner 2 to 4	66 (55.5%)	29 (50.0%)	37 (60.7%)	
Tanner 5	18 (15.1%)	9 (15.5%)	9 (14.8%)	
BMI-SDS	0.98 [−0.03, 1.70]	1.06 [0.15, 1.71]	0.98 [−0.15, 1.75]	0.848
Overweight, n (%)	58 (48.7%)	30 (51.7%)	28 (45.9%)	0.525
Obesity, n (%)	26 (21.8%)	13 (22.4%)	13 (21.3%)	0.884
WHtR	0.51 [0.46, 0.57]	0.50 [0.46, 0.57]	0.51 [0.46, 0.56]	0.928
WHtR ≥ 0.5, n (%)	70 (58.8%)	33 (56.9%)	37 (60.7%)	0.677
Waist-to-hip ratio	0.89 [0.85, 0.95]	0.90 [0.86, 0.96]	0.87 [0.84, 0.92]	0.010
DXA-derived body composition				
FM TBLH (kg)	17.6 [13.1, 23.8]	17.9 [12.1, 25.5]	17.6 [13.2, 23.4]	0.936
FM TBLH (%)	44.3 [35.7, 49.9]	41.0 [29.7, 48.4]	45.8 [41.0, 50.2]	0.002
FM trunk (kg)	8.5 [5.6, 11.4]	8.6 [5.2, 12.2]	8.3 [6.5, 10.9]	0.882
FM trunk (%)	42.4 [33.5, 48.9]	38.4 [26.6, 48.3]	44.1 [39.3, 49.3]	0.012
LM TBLH (kg)	23.5 [18.2, 31.8]	27.1 [19.7, 37.5]	21.5 [16.4, 26.6]	<0.001
FLR	0.79 [0.56, 0.99]	0.69 [0.42, 0.94]	0.85 [0.69, 1.01]	0.002
Biochemical parameters and blood pressure				
Triglycerides (mg/dL)	75 [58, 102]	72 [59, 107]	82 [54, 101]	0.640
Total cholesterol (mg/dL)	174 [153, 191]	168 [149, 187]	178 [164, 194]	0.028
HDL-C (mg/dL)	54 [44, 64]	51 [43, 63]	57 [45, 66]	0.121
LDL-C (mg/dL)	104 [88, 119]	93 [86, 124]	109 [90, 119]	0.182
Insulin (µU/mL)	13.3 [7.0, 21.6]	13.3 [6.8, 23.7]	11.1 [7.0, 21.0]	0.562
Glucose (mg/dL)	87 [83, 93]	87 [82, 91]	87 [83, 94]	0.705
HbA1c (%)	5.19 [4.99, 5.47]	5.33 [5.02, 5.49]	5.10 [4.90, 5.33]	0.012
HOMA-IR	2.58 [1.36, 4.52]	2.71 [1.33, 4.43]	2.43 [1.33, 4.65]	0.604
SPISE	8.52 [6.73, 10.62]	8.37 [6.69, 10.61]	8.73 [6.59, 10.63]	0.503
AST (U/L)	22 [18, 26]	22 [18, 30]	22 [19, 26]	0.591
ALT (U/L)	16 [12, 21]	17 [12, 22]	16 [12, 21]	0.898
Systolic blood pressure (mmHg)	105 [99, 115]	109 [100, 118]	102 [99, 109]	0.013
Diastolic blood pressure (mmHg)	60 [55, 66]	60 [55, 66]	60 [55, 66]	0.964

Abbreviations: ALT, alanine aminotransferase; AST, aspartate aminotransferase; BMI, body mass index; FLR, fat-to-lean mass ratio; FM, fat mass; HbA1c, glycated hemoglobin; HDL-C, high-density lipoprotein cholesterol; HOMA-IR, homeostatic model assessment of insulin resistance; LDL-C, low-density lipoprotein cholesterol; LM, lean mass; SDS, standard deviation score; SPISE, Single-Point Insulin Sensitivity Estimator; TBLH, total body less head; WHtR, waist-to-height ratio. Note: Continuous variables are reported as median [25th, 75th percentile], and categorical variables as n (%). Overweight was defined as BMI-SDS ≥ +1 (inclusive of participants with obesity, defined as BMI-SDS ≥ +2).

**Table 4:** Anthropometric, body composition and metabolic profile according to BMI and central adiposity (WHtR ≥ 0.5).

	BMI SDS ≥ 2 (n = 26)	BMI SDS < 2 (n = 93)	*p* Value	WHtR ≥ 0.5 (n = 70)	WHtR < 0.5 (n = 49)	*p* Value
Demographic, anthropometric and pubertal characteristics						
Age at diagnosis (years)	5.6 [3.1, 9.0]	7.6 [3.8, 10.1]	0.203	5.7 [3.1, 8.9]	8.7 [5.1, 10.9]	0.004
Age at follow-up (years)	13.1 [8.8, 15.1]	12.3 [9.6, 14.9]	0.994	11.2 [8.7, 14.3]	13.5 [11.1, 15.3]	0.035
Male sex, n (%)	13 (50.0%)	45 (48.4%)	0.884	33 (47.1%)	25 (51.0%)	0.677
Tanner 1	7 (26.9%)	28 (30.1%)	0.951	24 (34.3%)	11 (22.4%)	0.377
Tanner 2 to 4	15 (57.7%)	51 (54.8%)		36 (51.4%)	30 (61.2%)	
Tanner 5	4 (15.4%)	14 (15.1%)		10 (14.3%)	8 (16.3%)	
Height SDS	0.10 [−2.13, 1.22]	−0.60 [−1.60, 0.25]	0.224	−0.34 [−1.61, 0.87]	−0.50 [−1.65, 0.20]	0.371
BMI-SDS	2.62 [2.42, 3.15]	0.58 [−0.25, 1.24]	<0.001	1.45 [0.81, 2.46]	0.13 [−0.64, 0.82]	<0.001
Overweight, n (%)	-	-	-	51 (72.9%)	7 (14.3%)	<0.001
Obesity, n (%)	-	-	-	25 (35.7%)	1 (2.0%)	<0.001
WHtR	0.60 [0.56, 0.67]	0.49 [0.45, 0.53]	<0.001	0.56 [0.52, 0.61]	0.45 [0.43, 0.47]	<0.001
WHtR ≥ 0.5, n (%)	25 (96.2%)	45 (48.4%)	<0.001	-	-	-
Waist-to-hip ratio	0.90 [0.85, 0.99]	0.88 [0.85, 0.93]	0.229	0.91 [0.86, 0.97]	0.86 [0.83, 0.89]	<0.001
DXA-derived body composition						
FM TBLH (kg)	31.9 [22.9, 37.7]	15.8 [11.2, 20.9]	<0.001	20.8 [13.9, 31.5]	15.2 [9.3, 18.8]	<0.001
FM TBLH (%)	51.3 [46.6, 54.4]	41.3 [31.2, 46.5]	<0.001	47.9 [41.2, 51.5]	39.6 [28.4, 44.8]	<0.001
FM trunk (kg)	15.2 [9.8, 19.9]	7.4 [4.9, 9.4]	<0.001	9.9 [7.0, 15.2]	6.6 [3.4, 8.6]	<0.001
FM trunk (%)	50.3 [45.1, 54.8]	40.2 [29.3, 45.9]	<0.001	46.5 [41.2, 50.3]	36.6 [28.4, 41.2]	<0.001
LM TBLH (kg)	27.4 [22.3, 37.9]	21.1 [17.3, 28.6]	0.003	26.4 [20.7, 36.6]	25.6 [20.5, 33.1]	0.884
FLR	1.06 [0.87, 1.19]	0.71 [0.46, 0.87]	<0.001	0.92 [0.70, 1.06]	0.66 [0.39, 0.81]	<0.001
Biochemical parameters and blood pressure						
Triglycerides (mg/dL)	115 [84, 174]	70 [53, 94]	<0.001	94 [69, 126]	66 [53, 81]	<0.001
Total cholesterol (mg/dL)	182 [161, 195]	173 [153, 188]	0.134	176 [153, 191]	173 [156, 190]	0.719
HDL-C (mg/dL)	44 [35, 61]	55 [47, 64]	0.028	52 [41, 63]	56 [48, 64]	0.045
LDL-C (mg/dL)	115 [93, 127]	100 [87, 116]	0.035	105 [89, 121]	99 [86, 118]	0.483
Insulin (µU/mL)	20.5 [13.8, 36.1]	10.5 [6.1, 17.6]	0.001	16.1 [7.9, 27.1]	9.4 [6.3, 16.2]	0.024
Glucose (mg/dL)	86 [82, 91]	88 [83, 93]	0.487	88 [84, 93]	86 [82, 92]	0.451
HbA1c (%)	5.26 [5.02, 5.55]	5.17 [4.95, 5.41]	0.103	5.15 [4.92, 5.48]	5.22 [5.02, 5.39]	0.276
HOMA-IR	4.28 [2.72, 6.48]	2.13 [1.25, 3.89]	0.001	3.29 [1.69, 5.89]	2.02 [1.31, 3.49]	0.014
SPISE	4.86 [4.14, 6.83]	9.14 [7.72, 10.85]	<0.001	7.08 [5.06, 8.97]	10.09 [8.78, 12.16]	<0.001
AST (U/L)	23 [20, 33]	21 [18, 26]	0.064	22 [19, 27]	20 [17, 26]	0.137
ALT (U/L)	24 [16, 38]	15 [12, 19]	<0.001	18 [14, 26]	15 [11, 18]	0.002
Systolic blood pressure (mmHg)	106 [100, 118]	105 [99, 114]	0.231	106 [100, 118]	104 [96, 110]	0.042
Diastolic blood pressure (mmHg)	66 [58, 70]	60 [55, 65]	0.011	64 [58, 70]	59 [54, 63]	0.002

Abbreviations: ALT, alanine aminotransferase; AST, aspartate aminotransferase; BMI, body mass index; FLR, fat-to-lean mass ratio; FM, fat mass; HbA1c, glycated hemoglobin; HDL-C, high-density lipoprotein cholesterol; HOMA-IR, homeostatic model assessment of insulin resistance; LDL-C, low-density lipoprotein cholesterol; LM, lean mass; SDS, standard deviation score; SPISE, Single-Point Insulin Sensitivity Estimator; TBLH, total body less head; WHtR, waist-to-height ratio. Note: Continuous variables are reported as median [25th, 75th percentile] and categorical variables as n (%). Overweight is defined as BMI-SDS ≥ +1 (inclusive of participants with obesity, defined as BMI-SDS ≥ +2).

**Table 5:** SPISE values according to tumor categories, treatments, and hormone deficiencies.

Category	Subgroups	SPISE	*p* Value
Tumor histology			
Tumor type	Glial tumors (n = 39)	7.73 [6.10, 10.03]	0.001
	Embryonal tumors (n = 32)	10.44 [8.78, 12.28]	
	Craniopharyngioma (n = 17)	6.95 [6.17, 8.79]	
	Germ cell tumors (n = 16)	8.39 [6.36, 9.42]	
	Ependymoma (n = 12)	8.94 [8.12, 11.91]	
	Choroid plexus tumors (n = 2)	5.52 *	
	GH-secreting pituitary adenoma (n = 1)	**	
Hypothalamic–pituitary treatments			
Treatments involving the hypothalamic–pituitary region	None (n = 34)	8.72 [6.89, 11.85]	0.002
	Surgery only (n = 8)	7.47 [6.14, 8.76]	
	Radiotherapy only (n = 51)	9.62 [7.73, 10.86]	
	Surgery + radiotherapy (n = 26)	6.93 [4.82, 8.64]	
Hypothalamic–pituitary involvement			
Tumoral mass involving the hypothalamic–pituitary region	Yes (n = 62)	7.24 [6.30, 8.99]	<0.001
	No (n = 57)	9.93 [7.92, 12.01]	
Presence of hydrocephalus	Yes (n = 63)	8.84 [7.03, 11.40]	0.221
	No (n = 56)	8.36 [6.36, 10.23]	
Post-treatment pituitary deficiencies			
Hormone deficiency number after therapies	0 (n = 23)	8.98 [7.66, 12.33]	0.013
	1 or 2 (n = 61)	9.26 [6.91, 10.72]	
	3 or more (n = 35)	7.24 [6.38, 8.76]	

Note: Continuous variables are reported as median [25th, 75th percentile]. * IQR is not reported for choroid plexus tumors because only two patients were included. ** Values are not reported for GH-secreting pituitary adenoma because only one participant was included.

**Table 6:** Anthropometric, body composition and metabolic profile according to SPISE value.

	All (n = 119)	SPISE ≤ 7.82 (n = 48)	SPISE > 7.82 (n = 71)	*p* Value
Demographic, anthropometric and pubertal characteristics				
Age at diagnosis (years)	7.1 [3.8, 10.0]	7.9 [3.5, 11.1]	6.5 [3.8, 9.8]	0.636
Age at follow-up (years)	12.7 [9.5, 14.9]	13.7 [10.3, 16.0]	11.8 [9.4, 14.2]	0.018
Male sex, n (%)	58 (48.7%)	25 (52.1%)	33 (46.5%)	0.548
Tanner 1	35 (29.4%)	10 (20.8%)	25 (35.2%)	0.145
Tanner 2 to 4	66 (55.5%)	28 (58.3%)	38 (53.5%)	
Tanner 5	18 (15.1%)	10 (20.8%)	8 (11.3%)	
Height SDS	−0.40 [−1.60, 0.60]	−0.39 [−1.59, 1.05]	−0.47 [−1.63, 0.10]	0.235
BMI-SDS	0.98 [−0.03, 1.70]	2.06 [1.26, 2.71]	0.21 [−0.50, 0.98]	<0.001
Overweight, n (%)	58 (48.7%)	43 (89.6%)	15 (21.1%)	<0.001
Obesity, n (%)	26 (21.8%)	24 (50%)	2 (2.8%)	<0.001
WHtR	0.51 [0.46, 0.57]	0.56 [0.52, 0.61]	0.47 [0.45, 0.51]	<0.001
WHtR ≥ 0.5, n (%)	70 (58.8%)	45 (93.8%)	25 (35.2%)	<0.001
Waist-to-hip ratio	0.89 [0.85, 0.95]	0.91 [0.86, 0.96]	0.88 [0.84, 0.93]	0.028
DXA-derived body composition				
FM TBLH (kg)	17.6 [13.1, 23.8]	27.2 [20.8, 33.6]	13.7 [9.4, 17.6]	<0.001
FM TBLH (%)	44.3 [35.7, 49.9]	49.1 [44.2, 53.8]	40.8 [30.8, 45.5]	<0.001
FM trunk (kg)	8.5 [5.6, 11.4]	12.7 [10.1, 17.2]	6.3 [4.1, 8.4]	<0.001
FM trunk (%)	42.4 [33.5, 48.9]	48.4 [43.9, 54.1]	37.6 [26.7, 42.7]	<0.001
LM TBLH (kg)	23.5 [18.2, 31.8]	27.7 [22.7, 37.9]	19.9 [16.4, 26.8]	<0.001
FLR	0.79 [0.56, 0.99]	0.97 [0.79, 1.16]	0.69 [0.44, 0.84]	<0.001
Biochemical parameters and blood pressure				
Triglycerides (mg/dL)	75 [58, 102]	106 [82, 162]	65 [51, 82]	<0.001
Total cholesterol (mg/dL)	174 [153, 191]	173 [158, 193]	174 [153, 188]	0.466
HDL-C (mg/dL)	54 [44, 64]	46 [38, 58]	58 [49, 67]	<0.001
LDL-C (mg/dL)	104 [88, 119]	109 [93, 125]	94 [84, 115]	0.004
Insulin (µU/mL)	13.3 [7.0, 21.6]	17.8 [11.1, 27.9]	9.4 [5.8, 16.2]	<0.001
Glucose (mg/dL)	87 [83, 93]	85 [82, 91]	88 [83, 94]	0.270
HbA1c (%)	5.19 [4.99, 5.47]	5.20 [4.98, 5.49]	5.19 [4.99, 5.43]	0.820
HOMA-IR	2.58 [1.36, 4.52]	3.78 [2.25, 6.12]	2.04 [1.23, 3.57]	<0.001
AST (U/L)	22 [18, 26]	22 [18, 29]	22 [19, 26]	0.845
ALT (U/L)	16 [12, 21]	20 [13, 35]	15 [12, 18]	<0.001
Systolic blood pressure (mmHg)	105 [99, 115]	110 [100, 121]	103 [97, 109]	0.002
Diastolic blood pressure (mmHg)	60 [55, 66]	63 [58, 70]	60 [55, 65]	0.018

Abbreviations: ALT, alanine aminotransferase; AST, aspartate aminotransferase; BMI, body mass index; FLR, fat-to-lean mass ratio; FM, fat mass; HbA1c, glycated hemoglobin; HDL-C, high-density lipoprotein cholesterol; HOMA-IR, homeostatic model assessment of insulin resistance; LDL-C, low-density lipoprotein cholesterol; LM, lean mass; SDS, standard deviation score; SPISE, Single-Point Insulin Sensitivity Estimator; TBLH, total body less head; WHtR, waist-to-height ratio. Note: Continuous variables are reported as median [25th, 75th percentile], and categorical variables as n (%). Overweight was defined as BMI-SDS ≥ +1 (inclusive of participants with obesity, defined as BMI-SDS ≥ +2).

## Data Availability

Due to patient confidentiality, the study data are not publicly accessible. They are available from the corresponding author upon reasonable request.

## References

[1] Siegel R.L., Kratzer T.B., Giaquinto A.N., Sung H., Jemal A. (2025). Cancer statistics, 2025. CA Cancer J. Clin..

[2] Emery J., Butow P., Lai-Kwon J., Nekhlyudov L., Rynderman M., Jefford M. (2022). Management of common clinical problems experienced by survivors of cancer. Lancet.

[3] Brignardello E., Felicetti F., Castiglione A., Chiabotto P., Corrias A., Fagioli F., Ciccone G., Boccuzzi G. (2013). Endocrine health conditions in adult survivors of childhood cancer: The need for specialized adult-focused follow-up clinics. Eur. J. Endocrinol..

[4] Hammoud R.A., Mulrooney D.A., Rhea I.B., Yu C., Johnson J.N., Chow E.J., Ehrhardt M.J., Hudson M.M., Ness K.K., Armstrong G.T. (2024). Modifiable Cardiometabolic Risk Factors in Survivors of Childhood Cancer: JACC: CardioOncology State-of-the-Art Review. JACC Cardiooncol..

[5] Reisinger C., Nkeh-Chungag B.N., Fredriksen P.M., Goswami N. (2021). The prevalence of pediatric metabolic syndrome-a critical look on the discrepancies between definitions and its clinical importance. Int. J. Obes..

[6] Bolier M., de Winter D.T.C., Pluimakers V.G., Fiocco M., van den Berg S.A., Bresters D., van Dulmen-den Broeder E., van der Loo M.H.D., Höfer I., Janssens G.O. (2025). Prevalence and determinants of metabolic syndrome in 2338 childhood cancer survivors: A Dutch Childhood Cancer Survivor LATER 2 study. Cancer.

[7] Pluimakers V.G., van Waas M., Neggers S.J.C.M.M., van den Heuvel-Eibrink M.M. (2019). Metabolic syndrome as cardiovascular risk factor in childhood cancer survivors. Crit. Rev. Oncol. Hematol..

[8] Chemaitilly W., Li Z., Huang S., Ness K.K., Clark K.L., Green D.M., Barnes N., Armstrong G.T., Krasin M.J., Srivastava D.K. (2015). Anterior hypopituitarism in adult survivors of childhood cancers treated with cranial radiotherapy: A report from the St Jude Lifetime Cohort study. J. Clin. Oncol..

[9] McLaren D.S., Devi A., Kyriakakis N., Kwok-Williams M., Murray R.D. (2023). The impact of radiotherapy on the hypothalamo-pituitary axis: Old vs. new radiotherapy techniques. Endocr. Connect..

[10] Erfurth E.M., Müller H.L. (2025). Metabolic complications and their mechanisms in patients with craniopharyngioma. Best Pract. Res. Clin. Endocrinol. Metab..

[11] De Alcubierre D., Feola T., Puliani G., Ferrigno R., Amodeo M.E., D’aNiello F., Pirchio R., Sada V., Pandozzi C., Sesti F. (2025). Endocrine and metabolic consequences of childhood-onset craniopharyngioma during the transition age: A literature review by the TALENT study group. Rev. Endocr. Metab. Disord..

[12] Miao Y., Fan K., Peng X., Li S., Chen J., Bai R.N., Wei Y., Deng Y., Zhao C., Wu Q. (2023). Postoperative hypothalamic-pituitary dysfunction and long-term hormone replacement in patients with childhood-onset craniopharyngioma. Front. Endocrinol..

[13] van Iersel L., Mulder R.L., Denzer C., E Cohen L., A Spoudeas H., Meacham L.R., Sugden E., Meeteren A.Y.N.S.-V., Hoving E.W., Packer R.J. (2022). Hypothalamic-Pituitary and Other Endocrine Surveillance Among Childhood Cancer Survivors. Endocr. Rev..

[14] van Santen H.M., Müller H.L. (2025). Management of Acquired Hypothalamic Dysfunction and the Hypothalamic Syndrome; It Is More Than Obesity. Endocr. Rev..

[15] van den Oever S.R., Mulder R.L., Oeffinger K.C., A Gietema J., Skinner R., Constine L.S., Wallace W.H., Armenian S., Barnea D., Bardi E. (2025). Metabolic syndrome in childhood, adolescent, and young adult cancer survivors: Recommendations for surveillance from the International Late Effects of Childhood Cancer Guideline Harmonization Group. Eur. J. Endocrinol..

[16] Guolla L., Morrison K.M., Barr R.D. (2021). Adiposity in Survivors of Cancer in Childhood: How is it Measured and Why Does it Matter?. J. Pediatr. Hematol. Oncol..

[17] Kalyan A., Bradford M.C., Dubnicka A., Terrones I., Yao K., Runco D.V., Pihoker C., Baker K.S., Ronsley R. (2026). Body Composition Assessment and Correlation to Metabolic Profile in Survivors of Childhood Brain Tumors. J. Pediatr. Hematol. Oncol..

[18] Casirati A., Somaschini A., Muraca M., Cereda E., Morsellino V., Di Iorgi N., Caccialanza R., Haupt R. (2023). Fat-to-lean mass ratio as a tool to detect the dysmetabolic profile in childhood cancer survivors. Nutrition.

[19] Zong X., Kelishadi R., Hong Y.M., Schwandt P., Matsha T.E., Mill J.G., Whincup P.H., Pacifico L., López-Bermejo A., Caserta C.A. (2023). Establishing international optimal cut-offs of waist-to-height ratio for predicting cardiometabolic risk in children and adolescents aged 6–18 years. BMC Med..

[20] d’Annunzio G., Vanelli M., Pistorio A., Minuto N., Bergamino L., Iafusco D., Lorini R., Diabetes Study Group of the Italian Society for Pediatric Endocrinology and Diabetes (2009). Insulin resistance and secretion indexes in healthy Italian children and adolescents: A multicentre study. Acta Biomed..

[21] Paulmichl K., Hatunic M., Højlund K., Jotic A., Krebs M., Mitrakou A., Porcellati F., Tura A., Bergsten P., Forslund A. (2016). Modification and Validation of the Triglyceride-to-HDL Cholesterol Ratio as a Surrogate of Insulin Sensitivity in White Juveniles and Adults without Diabetes Mellitus: The Single Point Insulin Sensitivity Estimator (SPISE). Clin. Chem..

[22] Tantari G., Bassi M., Pistorio A., Minuto N., Napoli F., Piccolo G., La Valle A., Spacco G., Cervello C., D’annunzio G. (2024). SPISE INDEX (Single point insulin sensitivity estimator): Indicator of insulin resistance in children and adolescents with overweight and obesity. Front. Endocrinol..

[23] Ha J., Oh Y.R., Kang E., Nam H.K., Rhie Y.J., Lee K.H. (2022). Single Point Insulin Sensitivity Estimator for predicting type 2 diabetes mellitus in obese adolescents. Ann. Pediatr. Endocrinol. Metab..

[24] Barchetta I., Dule S., Bertoccini L., Cimini F.A., Sentinelli F., Bailetti D., Marini G., Barbonetti A., Loche S., Cossu E. (2022). The single-point insulin sensitivity estimator (SPISE) index is a strong predictor of abnormal glucose metabolism in overweight/obese children: A long-term follow-up study. J. Endocrinol. Investig..

[25] Song K., Lee E., Lee H.S., Lee H., Chae H.W., Kwon Y.J. (2025). Comparison of single-point insulin sensitivity estimator and other markers to predict metabolic syndrome in children and adolescents. Obes. Res. Clin. Pract..

[26] Louis D.N., Perry A., Reifenberger G., Von Deimling A., Figarella-Branger D., Cavenee W.K., Ohgaki H., Wiestler O.D., Kleihues P., Ellison D.W. (2016). The 2016 World Health Organization Classification of Tumors of the Central Nervous System: A summary. Acta Neuropathol..

[27] de Onis M., Onyango A.W., Borghi E., Siyam A., Nishida C., Siekmann J. (2007). Development of a WHO growth reference for school-aged children and adolescents. Bull. World Health Organ..

[28] Maffeis C., Banzato C., Talamini G., Obesity Study Group of the Italian Society of Pediatric Endocrinology and Diabetology (2008). Waist-to-height ratio, a useful index to identify high metabolic risk in overweight children. J. Pediatr..

[29] Zimmet P., Alberti K.G., Kaufman F., Tajima N., Silink M., Arslanian S., Wong G., Bennett P., Shaw J., Caprio S. (2007). The metabolic syndrome in children and adolescents—An IDF consensus report. Pediatr. Diabetes.

[30] Xi B., Zong X., Kelishadi R., Litwin M., Hong Y.M., Poh B.K., Steffen L.M., Galcheva S.V., Herter-Aeberli I., Nawarycz T. (2020). International Waist Circumference Percentile Cutoffs for Central Obesity in Children and Adolescents Aged 6 to 18 Years. J. Clin. Endocrinol. Metab..

[31] Gibson T.M., Mostoufi-Moab S., Stratton K.L., Leisenring W.M., Barnea D., Chow E.J., Donaldson S.S., Howell R.M., Hudson M.M., Mahajan A. (2018). Temporal patterns in the risk of chronic health conditions in survivors of childhood cancer diagnosed 1970–99: A report from the Childhood Cancer Survivor Study cohort. Lancet Oncol..

[32] Meacham L.R., Sklar C.A., Li S., Liu Q., Gimpel N., Yasui Y., Whitton J.A., Stovall M., Robison L.L., Oeffinger K.C. (2009). Diabetes mellitus in long-term survivors of childhood cancer. Increased risk associated with radiation therapy: A report for the childhood cancer survivor study. Arch. Intern. Med..

[33] Temtanakitpaisan Y., Saengnipanthkul S. (2022). Monitoring of Metabolic Syndrome and Cardiovascular Disease in Childhood Cancer Survivors. J. Adolesc. Young Adult Oncol..

[34] Hao H., Su Y., Feng M. (2024). Association Between Metabolic and Obesity Phenotypes and Diabetes Risk in Children and Adolescents. Diabetes Metab. Syndr. Obes..

[35] Romano A., Masino M., Rivetti S., Mastrangelo S., Attinà G., Maurizi P., Ruggiero A. (2024). Anthropometric parameters as a tool for the prediction of metabolic and cardiovascular risk in childhood brain tumor survivors. Diabetol. Metab. Syndr..

[36] van Schaik J., van Roessel I.M.A.A., Schouten-van Meeteren N.A.Y.N., van Iersel L., Clement S.C., Boot A.M., der Grinten H.L.C.-V., Fiocco M., Janssens G.O., van Vuurden D.G. (2021). High Prevalence of Weight Gain in Childhood Brain Tumor Survivors and Its Association with Hypothalamic-Pituitary Dysfunction. J. Clin. Oncol..

[37] Tuli G., Munarin J., Ragazzi P., Biasin E., Vallero S.G., Bertin D., Peretta P., De Sanctis L., Fagioli F. (2026). Endocrine disorders after treatment for pediatric brain tumors: Long-Term outcomes and risk factors. J. Clin. Endocrinol. Metab..

[38] Zaitoon H., Eyal O., Yackobovitch-Gavan M., Regev R., Issan Y., Roth J., Constantini S., Lebenthal Y., Brener A. (2025). Insights into body composition in pediatric craniopharyngioma patients after surgical treatment. Ther. Adv. Endocrinol. Metab..

[39] Song K., Lee E., Lee H.S., Youn Y.H., Baik S.J., Shin H.J., Chae H.W., Lee J.-W., Kwon Y.-J. (2025). Identification of pediatric MASLD using insulin resistance indices. JHEP Rep..

